# Bioactive luting cements on the adhesive interface of fiber post and root dentin: a comparative study of bond strength, water interaction, and enzymatic degradation

**DOI:** 10.1590/1678-7765-2025-0576

**Published:** 2026-03-02

**Authors:** Tiago Carvalho dos Santos, Giovanna Speranza Zabeu, Ana Carolina Almeida Lima, Mylena Proença Costa, Marcela Rocha Carrilho, Rafael Francisco Lia Mondelli, Linda Wang

**Affiliations:** 1 Universidade de São Paulo Faculdade de Odontologia de Bauru Departamento de Dentística, Endodontia e Materiais Odontológicos Bauru SP Brasil Universidade de São Paulo, Faculdade de Odontologia de Bauru, Departamento de Dentística, Endodontia e Materiais Odontológicos, Bauru, SP, Brasil.; 2 Universidade de São Paulo Faculdade de Odontologia de Bauru Departamento de Cirurgia, Estomatologia, Patologia e Radiologia Bauru SP Brasil Universidade de São Paulo, Faculdade de Odontologia de Bauru, Departamento de Cirurgia, Estomatologia, Patologia e Radiologia, Bauru, SP, Brasil.; 3 Midwestern University College of Dental Medicine Illinois IL USA Midwestern University, College of Dental Medicine, Illinois/ IL, USA.

**Keywords:** Bioactivity, Dental cements, Sorption and solubility, Tensile strength, Zymography

## Abstract

Innovative self-etching dental cements have been considered a versatile material with recent formulations incorporating bioactive additives.

**Objective:**

This study analyzed the role of these ingredients in systems used for luting intracanal posts.

**Methodology:**

Bond strength (BS), water sorption (WS), water solubility (WSB), and *in situ* zymography (IZ) were assessed. Three cements were tested: RelyX U200 (RU, bioactive-free control), RelyX Luting Plus (RL, fluoride-containing glass-ionomer), and BeautiCem SA (BC, S-PRG-based). Bond strength was evaluated in bovine root dentin (n=10) at the cervical, middle, and apical thirds at 7 days and 6 months. WS/WSB were assessed using 10 cement disks (10×1 mm) cycled through deionized water immersion (m1), desiccation (m2), and a second immersion (m3). For IZ, the specimens (n=3) were sectioned and incubated with fluorescein-conjugated gelatin for 48h at 37°C at both storage times (7 days and 6 months) for analysis by Confocal Laser Scanning Microscopy. Fluorescence intensity was quantified using Image J. Data were analyzed using ANOVA and Tukey’s test (p<0.05).

**Results:**

RU presented the highest BS and the lowest WS/WSB, while RL showed the opposite trend. BC demonstrated intermediate performance with no statistical differences from the other materials. For all cements, the cervical third and the 6-month period yielded the highest BS values. WS/WSB were greater for the bioactive cements (BC, RL). IZ results aligned with BS findings, revealing suppressed enzymatic activity over time. Based on these results, it can be concluded that RU exhibited superior mechanical stability despite lacking bioactivity.

**Conclusion:**

Among bioactive cements, BC emerged as an interesting alternative, offering improved bond strength, water stability, and bioactive potential compared to RL.

## Graphical abstract

**Figure:**
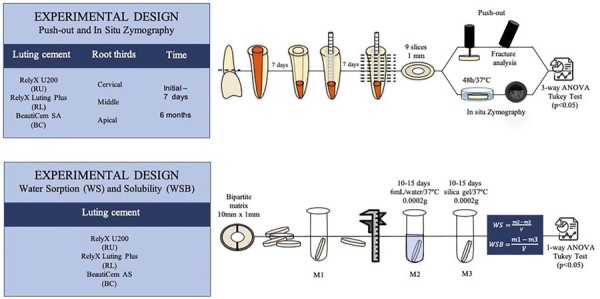

## Introduction

The concept of minimally invasive dentistry is fundamentally supported by the progress in understanding biological principles, which drives the innovative development of materials and techniques. The maintenance of pulp vitality is the goal, but in the case of non-vital teeth, endodontic treatment is no longer synonymous with a merely mechanical complexity issue. In contrast, efforts to explore and address the organic and inorganic components of the hard tissues in the root canal support the development of novel strategies to improve the long-term outcomes of endodontically treated teeth.^1-3^

Besides, the balance between convenience and cost-effectiveness is also relevant to determine the most appropriate treatment, including the endodontic and post systems approaches.^2,4,5^ Therefore, different options were introduced in the market offering benefits, but some of them showed drawbacks that limited their use.^2,6,7^ In this perspective, the use of fiberglass posts and adhesive luting cements became popular, mainly to reduce catastrophic failures associated with the metal post options.^6,7^ Also, since professionals are willing to work with direct procedures, companies and researchers have been encouraged to develop more feasible strategies.

Debonding has become a less frequent issue since the understanding of the non-compliance of acidic ingredients of dentin bonding systems with the luting systems has clarified the mechanisms behind early degradation of the interface.^8^ In this process, by-products stimulate the dentin host proteolytic enzymes, which have proven their role in compromising the quality of the adhesive interface. The actions of matrix metalloproteinases (MMPs) and cysteine cathepsins (CCs) have driven the innovation toward strategies focused on their inhibition under relevant physical–mechanical conditions.^1,9,10^ Inhibitory agents such as chlorhexidine have been extensively investigated for this purpose and there is evidence of their effectiveness, even if it is reduced over time.^1,9,10^ However, the major obstacle resides in the need for an additional step, which is certainly hard to approach in root canal.

Self-adhesive luting cements represent a revolutionary contemporary category.^11-13^ In particular, the elimination of the use of dentin bonding systems, which can exacerbate the vulnerable interface, seems to be interesting.^14-15^Beyond the less sensitive technique and good bonding ability, the addition of bioactive ingredients has been proposed.

The term bioactivity varies according to the tissue to be treated and their function. According to Vallittu, et al.^16^ (2018), antimicrobial and adhesive properties are mandatory to classify a material as bioactive. RelyX Luting is a glass ionomer-based self-etching system with proven fluoride release.^17^ More recently, companies have engaged in developing resin-based materials with novel technologies, such as a xerogel introduced by Shofu Inc. The S-PRG technology is a Surface Pre-Reactive Glass preactivated by polysiloxane acid constituted by a trilaminar particle. As this surface is intended to be porous and preactivated, once in contact with the oral environment, it is able to release multiple ions to avoid demineralization and strengthen the dental tissues.^18-20^ This pool of ions includes sodium, strontium, aluminium, silicate, boron, and fluoride. In the case of resin-based materials, they are silanized fillers. These ingredients can stimulate benefits and induce positive cellular responses.^16^ Since endodontically treated teeth face challenges related to sanitizing protocols, instrumentation and luting techniques with bioactive properties are very welcome to reduce their inherent limitations.

For bioactive actions, water is essential, in particular when ions are involved, which is the case of self-adhesive luting cements based on acidic functional monomers.^21,22^ When bioactive ingredients are added, the ionic elements increase this potential. Therefore, the balance of a moist tissue is mandatory to start the reaction and avoid early degradation. In this context, the water sorption and water solubility are two important related properties associated with the bonding capacity. To understand details of this interface and considering the action of enzymes, the zymography assessment is also interesting.

Thus, this study aimed to investigate the performance of self-adhesive luting cements regarding their physical, mechanical, and biological properties. The null hypotheses were that there would be no difference between treatments for bonding strength (push out), water sorption, water solubility, and *in situ* zymography assessments.

## Methodology

**Figure 1:**
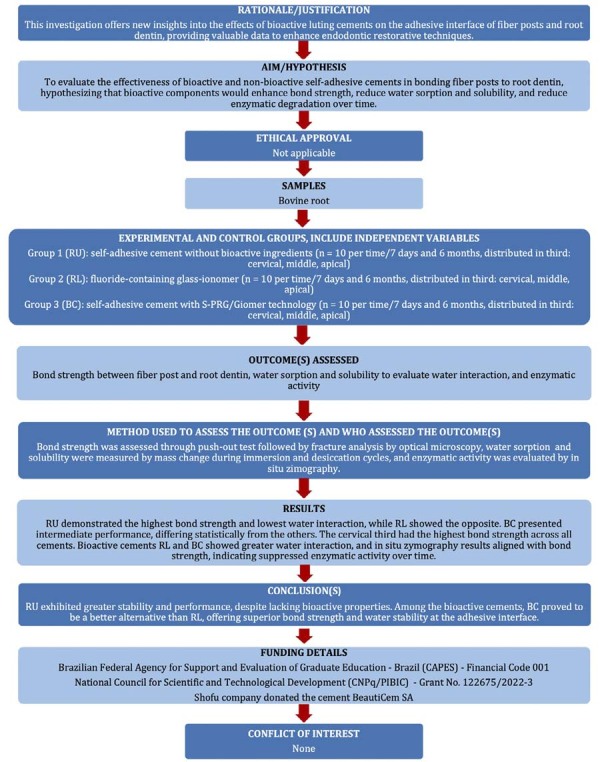
Flowchart written according PRILE 2021 guidelines.

The manuscript of this laboratory study has been written according to the Preferred Reporting Items for Laboratory studies in Endodontology (PRILE) 2021 guidelines (Figure 1).

### Experimental design

This *in vitro*, parallel study was performed by a single operator under blinded conditions during the tests. For specimen preparation, two pre-calibrated operators conducted all the steps to ensure maximum standardization of the experimental protocols.

**Figure 2:**
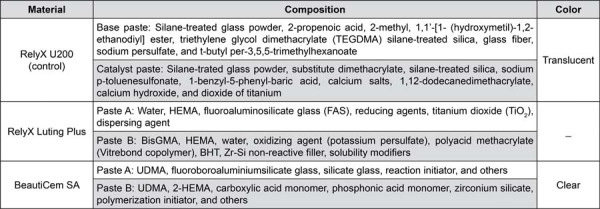
Materials, compositions, and application mode.

The tested materials are presented in Figure 2.

HEMA= Hydroxy ethyl methacrylate; BisGMA= Bisphenol A glycidyl methacrylate; UDMA= urethane dimethacrylate.

### Sample size calculation

To calculate the sample size, previous pilot studies were performed. Based on the use of G*Power 3.1 software (Aichach, Germany), the values of α=0.05 and power (1-β)=0.8 were considered. Therefore, it was determined a total of 10 for BS, 10 for WS/WSB, and 3 for IZ tests.

### Bonding test (push out)

For this test, the experimental design involved 3 factors: luting cement systems on 3 levels (RelyX U200 [RU], RelyX Luting 2 [RL], and BeautiCem SA [BC]), root thirds on 3 levels (cervical, middle, and apical) and storage time on 2 levels (7 days and 6 months). The main response variable was the bond strength to radicular dentin by *push-out* test followed by fracture analysis by optical microscopy, according to the recommendations of ISO 11405:2015 standards.

### Specimen preparation

Sixty straight bovine roots were selected and stored in 0.1% thymol solution in supersaturated saline solution at 4°C, which was renewed weekly. The teeth were sectioned at the cementoenamel junction using a double-diamond faced disc on a cutting machine (Isomet, Buehler, Lake Bluff, IL, USA) with constant irrigation to obtain 17mm- root lengths, measured from the cervical region to the apex. Rounded root anatomy was preferred, and the specimens were randomized into the groups. The endodontic access was established with a working length of 16mm. The step-back technique was employed to prepare all the roots with K-type files (Maillefer-Dentsply, Ballaigues, Switzerland) up to the ISO standard size 45. Before and after the endodontic procedures, the canal was irrigated with deionized water to avoid dentinal tubule obliteration. Although commonly used, hypochlorite-based products were avoided to minimize interference with polymerization. After the instrumentation, the irrigation was conducted with EDTA (Biodinâmica, Ibiporã, PR, Brazil), which was subsequently washed with deionized water and dried with absorbent paper cones (Tanari, Manacapuru, AM, Brazil). Following, gutta percha tips (Tanari, Manacapuru, AM, Brazil) associated with calcium hydroxide-based sealers (Sealer 26 – Dentsply, Rio de Janeiro, RJ, Brazil) were used to treat the canals based on the lateral condensation technique. After 7 days of storage in deionized water at 37°C, the root canals were unfilled with Gates-Glidden #2 drill. Each canal was extended with a low rotation drill according to the post diameter. The depth measurement of the post from the cementoenamel junction was 13mm with a 1.5mm diameter, resulting in 3mm of apical sealing. The specimens were randomized (n=10) according to the cement luting agent (Figure 2). Before luting, all the specimen roots were covered with aluminum foil, minimizing the impact of external lighting.

For the preparation of the fiber post (Reforpost, Ângelus, Londrina, PR, Brazil), 37% phosphoric acid was applied to clean the post (Condac, FGM, Joinville, SC, Brazil) for 15 seconds and washed for the same time. After drying with an air jet, silane was applied (Primer Silano, Ângelus, Londrina, PR, Brazil) and left to react for 5 minutes. In terms of canal preparation and cementation, the operator was previously calibrated for the pretreatment procedures. Initially, the specimen was irrigated with deionized water and dried with absorbent paper cones of a caliber compatible with the canal diameter. Subsequently, the cements were inserted into the root canal using a Centrix type syringe with thin metallic tip. The posts were seated into the root canal, reducing the air bubble incorporation risk. The excess cement was removed with a micro-brush and then light-cured for 40 seconds using Radii-Cal (SDI, Bayswater, Australia) with 1,000 mW/cm^2^ irradiance. Three 1mm-thick sections per third were obtained: cervical, middle, and apical, totalizing 9 sections per root. After 24 hours, the specimens were sectioned perpendicularly to the long axis under refrigeration (Isomet, Buehler, Lake Bluff, IL, USA). The specimens were stored in artificial saliva at 37°C for 7 days or 6 months.

### Push-out test and interface fracture analysis

Bond strength was assessed by a push-out test using a universal test machine (Instron 3342 – Instron, Canton, MA, USA). The most coronal surface of each slice was positioned downwards, on the base of the equipment, and the post was centered over the opening of a metal platform to allow the post to move without offering resistance opposite to the load application, which was applied at 0.5mm/min in coronal-apical direction. The diameter of the application tip varied from 0.8 to 1.3mm, avoiding contact with the internal dentin walls during testing. The values were recorded in KgF and converted into MPa. The specimen thickness was measured with a digital caliper (Mitutoyo digital caliper, Mitutoyo America, Aurora, IL, USA) and the adhesion surface was calculated according to the formula of a cone shape: A = π (R2+R1) [h^2^ + (R2-R1)^2^]0.5, where R1 = base radius, R2 = top radius, and h = height. All the specimens were analyzed in optical microscope (50× and 200× magnification) (DINO-LITE plus digital microscope, AnMoEletronics Corporation Hsinchu, China) to determine the fracture type. The fractures were categorized as: A C/D (adhesive between cement and dentin), A C/P (adhesive between cement and post), CP (cohesive on the post), CC (cohesive on the cement), and M (mixed).

### Statistical analysis

The data was statistically analyzed with the Statistica software (Statsoft, Tulsa, OK, USA). The assumption of normal distribution and variation equality were verified for all the variables by the Kolmogorov-Smirnov and Levene’s tests, respectively. Since the premises were satisfying, the data was subject to ANOVA test by 3-criteria (p<0.05). For individual comparisons, Tukey’s test was used (p<0.05).

### Water sorption and water solubility tests

#### Specimen preparation

The tested cements were prepared as disks (n=10, 10×1mm), according to ISO 4049:2000 specifications for water sorption and water solubility tests. At the base and top, polyester strips were used and pressed with a glass coverslip, allowing the extravasation of excess material, aiming to obtain a regular surface and to avoid contact with oxygen. Each surface was polymerized for the recommended time according to the manufacturer using Radii-Cal (SDI, Bayswater, Victoria, Australia) with 1,000mW/cm^2^ irradiance.

The specimens were stored in containers with silica gel at 37°C (Synth, Blue Mesh 2-4kmm, São Paulo, SP, Brazil). The disks were tested in a standardized way using an analytical balance (GR-202, A & D Engineering, Inc., San Jose, CA, USA) with 0.01mg readability, obtaining a constant mass value (M1) without loss of water (oscillation 0.0002g). After that, the specimens were stored in distilled water at 37°C for approximately 10 days in 6mL, until new stabilization. Before weighing, each specimen was carefully dry in a standardized way with a paper towel. After new mass stabilization, the M2 value was recorded. A second drying cycle was performed in which the specimens were subjected to another drying process for about 10 days, until a new stabilization phase was reached, thus yielding the value of M3. The variation limit was 0.0002g.^22,23^ The water sorption and water solubility were calculated (μg/mm^3^) using the following equations:

## Statistical analysis

The assumptions of normal distribution and variance equality were verified for all variables by Kolmogorov-Smirnov and Levene’s tests, respectively. As the premises were satisfying, the data was subject to ANOVA in one-way criteria (p<0.05) for WS and WSB. For individual comparisons in case of statistical difference, Tukey’s test was applied (p<0.05).

## *In situ* zymography analysis

### Specimen preparation

The *in situ zymography* procedure was adapted from the protocols of Mazzoni, et al.^24^ (2012) and Speranza Zabeu, et al.^25^ (2023). Specimens (n=4) were sectioned into slices of approximately 1mm-thickness. For the 6-month evaluation, the slices were stored in artificial saliva at 37°C, with the solution refreshed every 15 days. They were prepared immediately before each zymographic analysis. The slices were immersed in 1% phosphoric acid (Sigma-Aldrich, Saint Louis, MO, USA) for 30 seconds, followed by a 60-second rinse in distilled water. Zymographic analysis was performed using a self-quenched fluorescein-conjugated gelatin (EnzChek gelatinase/collagenase assay kit, Molecular Probes, Eugene, OR, USA). The gelatin was diluted in a buffer solution (150 mM NaCl, 5 mM CaCl2, 50 mM Tris-HCl, pH 8.0) and mixed with an anti-fading agent (Mounting Medium with DAPI H-1200, Vectashield, Vector Laboratories LTD, Cambridgeshire, UK) at a 1:8 concentration. Each slice was placed on a culture plate, and 150 μL of the fluorescent gelatin mixture was applied, fully covering the adhesive/dentin interface. The samples were then incubated in a dark, humid chamber at 37°C for 48 hours. Following incubation, the interfaces were examined using a Confocal Laser Scanning Microscope (Leica TCS SPE, Leica Microsystems, Mannheim, BW, Germany) with excitation and emission peaks at 460 and 520 nm, respectively. Two images per sample were captured from 10 μm-thick optical sections at different focal planes. Fluorescence within the hybrid layer was visualized using the green channel, while reflectance was used to assess optical density. The images were then merged, and gelatinolytic activity was quantified using ImageJ software (Research Services Branch, National Institute of Mental Health, Bethesda, MD, USA). Four sequential images from each slice were qualitatively and quantitatively analyzed, with fluorescence intensity expressed in arbitrary units.

## Statistical analysis

Data were statistically analyzed using Statistica software (StatSoft, Tulsa, OK, USA). The assumption of normal distribution and variance homogeneity were verified for all variables using the Kolmogorov-Smirnov and Levene’s tests, respectively. As the assumptions were met, a three-way ANOVA was performed (p<0.05). For post hoc comparisons, Tukey’s test was applied (p<0.05).

## Results

### Push-out test and interface fracture analysis

The bond strength values for the initial period and 6-month evaluations are presented in Table 1. Statistical analyses revealed significant differences for all individual factors: material (p<0.00001), third (p=0.00112), and time (p=0.00251). However, none of the interactions between factors were statistically significant: cement×third (p=0.77429), cement×time (p=0.05099), third×time (p=0.64019), and cement×third×time (p=0.97794).

**Table 1:** Average values and standard deviation of push-out bond strength in the initial and 6-month time (MPa).

Luting Cements	Initial			6 months		
	Cervical	Middle	Apical	Cervical	Middle	Apical
RU	6.46 (3.02)^Aa*^	6.56 (3.00)^Ba*^	5.11 (2.17)^Ba*^	7.46 (1.25)^Aa+^	6.91 (2.91)^Ba+^	6.50 (2.88)^Ba+^
RL	2.60 (0.83)^Ac*^	1.70 (1.18)^Bc*^	1.16 (0.87)^Bc*^	4.39 (1.75)^Ac+^	2.92 (1.54)^Bc+^	3.32 (1.90)^Bc+^
BC	4.90 (1.31)^Ab*^	3.83 (1.34)^Bb*^	3.39 (1.77)^Bb*^	5.17 (1.47)^Ab+^	3.53 (2.07)^Bb+^	3.17 (1.72)^Bb+^

Capital letters represent statistically significant differences between columns for the same row for each time (third factor).

Lowercase letters represent statistically significant differences between rows in the same column for each time (resin cement factor).

Symbols represent statistically significant differences between times for each resin cement and third.

Regarding the luting cements, RU exhibited the highest bond strength values, followed by BC, while RL presented the lowest values. All groups were statistically different from each other. Concerning the root third factor, the cervical third showed the highest bond strength values, which were significantly different from those of the apical third. The middle third showed significant differences compared to either the cervical or apical thirds. For the time factor, the 6-month period yielded superior bond strength values compared to the initial evaluation.

**Figure 3:**
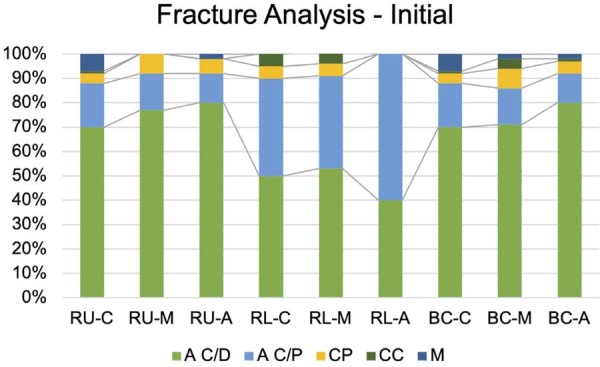
Failure mode from immediate time of specimens tested by push out: A C/D (adhesive between cement and dentin), A C/P (adhesive between cement and post), CP (cohesive on the post), CC (cohesive on the cement), M (mixed). A C/D type was predominant for RU and BC, while A C/P was notable for RL.

**Figure 4:**
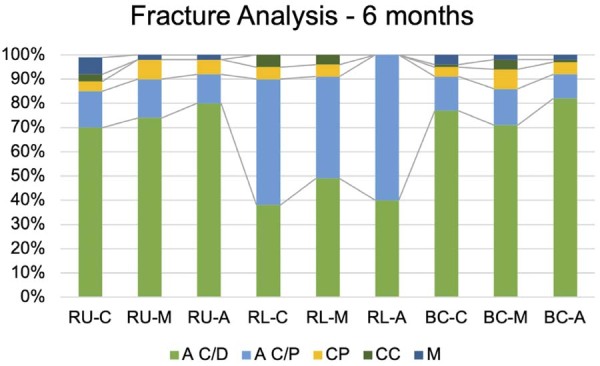
Failure mode from 6-month time of specimens tested by push out: A C/D (adhesive between cement and dentin), A C/P (adhesive between cement and post), CP (cohesive on the post), CC (cohesive on the cement), M (mixed). Similar to immediate time, A C/D type was predominant for RU and BC, while A C/P was notable for RL.

In the fracture analysis, the RU and BC cement groups predominantly exhibited adhesive failures at the cement-dentin interface, with adhesive failures between cement and post as the second most common mode. For RL, the predominant failure mode was adhesive failure between cement and post, followed by adhesive failure between cement and dentin. The fracture analyses are illustrated in Figures 3 and 4.

### Water sorption and water solubility

WS and WSB data are presented in Table 2, indicating that all luting cements had statistically significant differences (p<0.00001). For both tests, RU presented the lowest values, followed by BC, while RL demonstrated the highest values for both WS and WSB.

**Table 2:** Mean values and standard deviation of water sorption and solubility in the initial and 6-month periods (MPa).

Luting Cements			
	RU	RL	BC
WS	20.03 (2.51)^A^	205.75 (5.99)^C^	35.17 (5.25)^B^
WSB	-2.61 (0.61)^A^	25.65 (3.92)^C^	2.16 (2.58)^B^

Capital letters indicate statistical differences between columns for the same row.

### *In situ* zymography analysis

**Figure 5:**
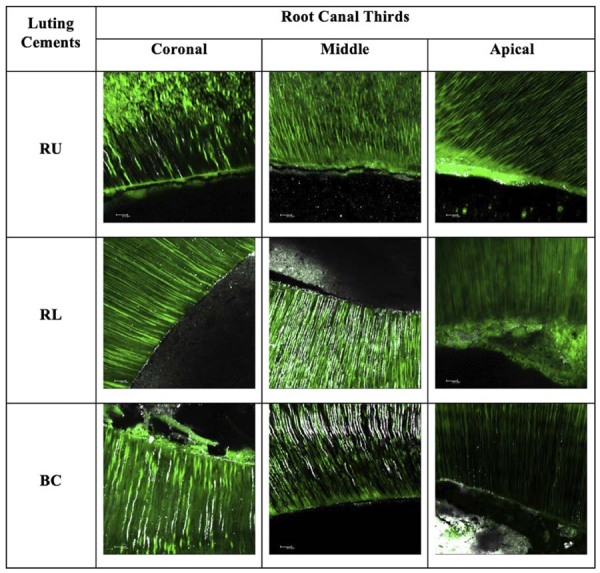
Initial time CLMS images showing that RU specimens indicated higher fluorescence, which corresponds to more intense enzymatic activity. For RL and BC, zymographic assessments indicated lower activity.

**Figure 6:**
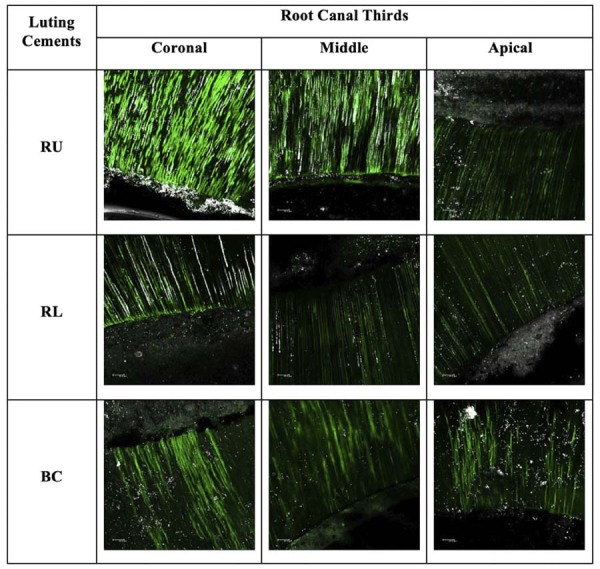
6-month CLMS images showing the same ranking seen at initial time, but with exacerbated intensity for RU. On the contrary, the luting cements with bioactive ingredients indicated the suppression of enzymatic activity, in particular for BC, denoting the role of bioactive ingredients.

Time as an isolated factor showed a statistically significant effect (p<0.00001). Significant differences were observed between the initial and 6-month evaluations. In the initial evaluation, no statistically significant differences were found between the luting cements across the cervical, middle, or apical regions. Nevertheless, after 6 months, all cements exhibited a significant increase in relative fluorescence units in each region. The data are summarized in Table 3, and representative images of the dentin-cement interface are presented in Figures 5 and 6.

**Table 3:** Mean values and standard deviation (arbitrary unit) of fluorescence in the hybrid layer in the initial and 6-month periods.

Luting Cements	Initial			6 months		
	Cervical	Middle	Apical	Cervical	Middle	Apical
RU	198.53 (17.05)^A^	195.98 (19.48)^A^	194.15 (40.81)^A^	218.34 (22.08)^B^	206.32 (9.06)^B^	239.89 (6.07)^B^
RL	209.79 (11.79)^A^	186.81 (27.03)^A^	197.35 (6.47)^A^	241.37 (7.89)^B^	240.15 (7.34)^B^	233.59 (5.75)^B^
BC	186.96 (49.18)^A^	204.50 (33.12)^A^	185.10 (38.30)^A^	207.09 (9.04)^B^	217.03 (7.22)^B^	216.74 (19.19)^B^

Capital letters represent statistically significant differences between columns within the same row across different time periods.

## Discussion

Until recent decades, research on endodontically treated teeth primarily focused on mechanical factors influencing their performance.^6,26^Additionally, the complex anatomy of root canals and the significance of the amount and quality of the remaining dental structure encouraged efforts to develop materials aimed at minimizing limitations in the restoration of these teeth.^6,27,28^

The introduction of adhesive technology revolutionized bonding to dentin and posts, significantly reducing catastrophic mechanical failures.^6,26^ Consequently, investigations on luting materials and techniques advanced, emphasizing their feasibility and versatility in addressing the clinical challenges of root canal treatments, particularly in achieving effective bonding at the apical third, where light penetration remains a critical limitation for resin-based cements reliant on monomer conversion.^29,30^ Interactions between luting materials and pretreatment agents, such as irrigation solutions, have also drawn attention due to the potential for adverse effects when these agents are used independently.^1,9^ These findings have prompted a focus on self-etching luting cements, which reduce procedural steps and thereby minimize technical errors. Such systems offer additional benefits, including protection against the activation of proteolytic enzymes and reduced dentin demineralization by eliminating the need for prior acid conditioning, instead promoting chemical interaction with the dentin.^1,30^ These advantages have demonstrated clinical efficacy.^30^

Glass ionomer and S-PRG/Giomer-based systems have been incorporated into resin-based agents, resulting in cements indicated for the luting purposes, including fiber post.^31,32^ Both systems provide elements that can be advantageous in biological contexts, offering conditions to resist degradation over time. Classically, the glass ionomer-based materials are classified as bioactive products, because of their ability to release fluoride.^33,34^ Recently, a boom in the use of S-PRG filled materials has highlighted the benefits of a technology capable of releasing multiple ions that can enhance the resistance of dentin and offer antimicrobial protection.^18,19^ In a previous publication, Yassen, et al.^35^ (2016) showed that a repair cement containing S-PRG was able to act as an antibacterial agent against endodontic pathogens. Also, S-PRG has been shown to inhibit metalloproteinases.^35,36^

Based on the overall results of the tests performed in this study, S-PRG-based luting cement seems to offer balanced benefits. All null hypotheses were rejected. The push-out bond strength test, a commonly employed method for assessing root canal treatments, revealed that resin-based luting cement (RU) achieved the highest bond strength, while the bioactive cement (BC) performed comparably. In contrast, the ionomer-based system (RL) yielded the lowest bond strength. These findings align with studies indicating that materials with higher hydrophilic content typically exhibit lower mechanical resistance.^31^

In addition to these findings, the increase in push-out bond strength observed after six months deserves further consideration. Although unexpected, this trend may be partially explained by the intrinsic characteristics of the tested materials. Self-adhesive luting cements contain hydrophilic functional monomers that initially promote ionic diffusion, water uptake, and interaction with dentin substrates. Over time, however, continued ion exchange and maturation of the polymer network may enhance micromechanical interlocking and chemical stability at the bonding interface. This behavior is consistent with the mechanism proposed by Collares, et al.^32^ (2015), who demonstrated that the long-term bond strength of a self-adhesive resin cement significantly increased due to its higher swelling coefficient, an effect attributed to water sorption, induced expansion, which improves adaptation to the root canal walls and reinforces interfacial retention. This phenomenon is particularly relevant for materials containing bioactive components, such as S-PRG fillers or glass ionomer phases, which can gradually release ions capable of strengthening the dentin structure and reducing enzymatic degradation.^18,35^ Collectively, these mechanisms help clarify the notable increase in bond strength at six months and reduce subjectivity in interpreting these results.

In this context, the analyzed results from water sorption and solubility tests further confirmed that BC and RL were more susceptible to water interaction, a feature necessary for activating their bioactive components, albeit potentially compromising mechanical properties to some extent.^22^So far, glass ionomer-based systems have been recognized by their valuable capacity to remineralize dental tissue and by their antibacterial potential.

The results regarding WS and WSB highlight the more susceptible performance of RL, which is a glass ionomer-based material. The significantly higher water sorption and solubility observed for RL can be attributed to its glass ionomer composition, which contains a greater proportion of hydrophilic polymer chains and reactive ionic components.^33-35^ These features facilitate ion exchange and fluoride release, reinforcing the material’s recognized bioactive potential; however, they also substantially increase its affinity for water.^34^ As a consequence, RL becomes more susceptible to water uptake and hydrolytic degradation, which may negatively influence its dimensional stability and mechanical integrity over time. Compared to the resin-based systems such as RU and BC, RL exhibits a trade-off in which enhanced bioactivity is accompanied by reduced mechanical robustness, a factor that should be considered when selecting luting agents for clinical applications that demand long-term stability. Considering the main properties analyzed, it is plausible to realize that S-PRG filled materials seem more promising in terms of physical-mechanical aspects.

Also, this study aimed to associate the assessment of physical-mechanical methods with *in situ* zymography to provide information regarding the interaction on the bonding interface related to the biological capacity to inhibit the proteolytic activity.^25,36,37^Based on this test, the results did not confirm the difference between the materials on each analyzed time. Nevertheless, it is interesting to note that the products containing the bioactive additives notably played a role in suppressing enzymatic activity after 6 months.

As these materials do not require previous acid conditioning before their application, this reduced exposure of collagen fibrils in their bonding mechanism was likely important in minimizing the initial condition that could activate MMPs. After 6 months, although no quantitative differences were observed, bioactive materials (BC and RL) showed a more visible reduction in fluorescence, suggesting that bioactive ingredients may positively contribute to the suppression of gelatinolytic activity. To confirm this, long-term evaluations are required.

While the combination of outcomes from the proposed tests is aligned, this study supports the idea that S-PRG is on the way to provide bioactive benefits without compromising mechanical performance. Considering the limitations of this study, we acknowledge that no mechanical loading was applied during storage, and no additional stimuli, such as microcosm biofilms or altered dentin substrates, were incorporated. However, this study was necessary to encourage new studies considering these factors. Future investigations should also evaluate the self-adhesive mode more comprehensively, examine the effects of canal acid conditioning, and further explore enzymatic activity under different clinical conditions. In addition, assessments extending beyond six months are recommended to better clarify the long-term stability and bioactive potential of these materials.

Overall, the results support the clinical application of all tested systems. BC, in particular, offers a balance of bioactive and mechanical properties, making it a versatile option for professionals, even though its bond strength may not match the highest-performing systems.

## Conclusions

The findings of this study support the application of a self-etching luting system containing S-PRG particles, which demonstrated a balanced combination of mechanical and biological benefits. RelyX U200 showed greater stability and performance, despite the absence of bioactive properties. Among the bioactive cements, BeautiCem was more stable than RelyX Luting Plus in terms of bond strength and water interaction. *In situ* zymography revealed that all systems suppressed enzymatic activity over time, particularly after six months, with bioactive cements showing a greater potential to reduce gelatinolytic activity. Long-term evaluations are recommended to confirm these findings. Overall, RelyX U200 offers superior mechanical stability, while BeautiCem emerges as a better bioactive alternative with optimal bond strength and water stability for clinical use.
